# Supplementary material for: Prediction and practical application of bauxite mineralization in Wuzhengdao area, Guizhou, China
Source: PLoS One. 2024 Jul 31;19(7):e0305917. doi: 10.1371/journal.pone.0305917 (PMC11290671; doi:10.1371/journal.pone.0305917)
Supplement: Attachment — Submitted filename: Response to Reviewers5.26.doc [file pone.0305917.s002.doc]

Dear editor：

Thank you very much for your letter. We have learned much from two reviewers’ comments, which are fair, encouraging and constructive. After carefully studying the comments and your advice, we have made corresponding changes. The main revisions are listed below.

# For reviewer one:

Reviewer #1: As the study analyzes the factors that influence/contribute to the potential of three different ML algorithms to produce accurate results when used to explore a mineral deposit. As discussed in the conclusion, each method has a different data structure. This difference has produced different results. On the other hand, it is not possible to test the success of these methods in a field that is still under exploration (reserve and metal content information is unknown). In this case, it would not be meaningful to compare the methods.In conclusion, although this study discusses the predictive efficiency of three different algorithms, there is no criterion (reserve and metal content information) to prove that each of them is appropriate or not. Therefore, there is no conclusion reached by the study. In other words, the appropriate methodology and/or the data set that should be used for a successful prospecting etc. are not proposed

Thanks to the opinions of the reviewer, The opinions of the reviewers are very precise and directly reveal the problems existing in our discipline, which is worthy of our deep consideration and change. Mineral prediction has always been called pseudoscience. Different models, different algorithms and different people will have different results in the same area, because there are too many boundary conditions in our geological conditions, and many results are unknown.

As the reviewer said, As discus sed in the conclusion, each method has a different data structure. This difference has produced different results”,“ it is not possible to test the success of these methods in a field that is still under exploration”Indeed, what we are doing is not precise work. This is different from the fine research in the field of materials metallurgy. Our work can only screen out relatively effective methods, which increases the probability of mineral exploration. This method is suitable for regions with the same geological background to find similar types of minerals. At the same time, we have had successful examples of application in the region. Our mineral exploration results have been submitted to the Department of Natural Resources of Guizhou Province and have been recognized by the country. Here is the approval given by our government as support, hoping to receive some recognition and recognition from the reviewers.

# For reviewer two:

1 The significance of the study is not well emphasized. This should be explained concisely.Grammar check is required all through the manuscript. Check page 13 line 58 for correction. A word appears incorrectly written.Page 13 line 72 should be prediction-based model ….Authors should explain why the sensitivity of logistic regression decreased to varying degrees

We add a paragraph highlighting the research implications.
This work can provide ideas for geological analogy and geological research in regional prospecting projects, can concentrate on summarizing complex geological phenomena into some data parameters, can provide a complete concept of metallogenesis, is conducive to the study of metallogenic environment and the distinction between metallogenic environment and non-metallogenic environment, and can formulate a reasonable and economic prediction scheme for regional prospecting; It is an indispensable link in the process of transformation from qualitative to quantitative research

We have invited professional English institutions to polish and revise the language of the article.

Yang Wu
